# Incidence of and Predictors for Early Return Visits to the Emergency Department: A Population-Based Survey

**DOI:** 10.1097/MD.0000000000001770

**Published:** 2015-10-30

**Authors:** Mingchung Ko, Yaling Lee, Chuchieh Chen, Pesus Chou, Dachen Chu

**Affiliations:** From the Department of Emergency Medicine and Surgery, Taipei City Hospital (MK, DC); Institute of Public Health and Community Medicine Research Center, National Yang-Ming University (MK, YL, PC, DC); Department of Health Care Management, National Taipei University of Nursing and Health Sciences (MK, CC, DC); Department of Dentistry, Taipei City Hospital (YL); and Department of Dentistry, School of Dentistry, National Yang-Ming University, Taipei, Taiwan (YL).

## Abstract

The aim of this study is to estimate the proportion of and predictors for early return visits (ERVs) to the emergency department (ED) in Taiwan.

This is a population-based study using data of 1 million people randomly selected from all beneficiaries of the Taiwan National Health Insurance. All ED visits in 2012 were analyzed. The ERVs to the ED were defined as those ED revisits within 3 days after the initial ED visit. We employed a generalized estimation equation model to investigate the independent effects of various characteristics associated with the initial ED visit on ERVs.

The overall proportion of ERVs within 3 days with a same dichotomous diagnostic category according to injury or noninjury was 4.3% (6740/158,132), and the overall proportion of hospitalizations after ERVs was 24.1% (1627/6740). Male subjects (4.3%) were more likely to have ERVs with an adjusted odds ratio (AOR) of 1.10 (95% confidence interval [CI]: 1.04–1.16). Compared with patients aged 18 to 64 years (4.0%), those aged >64 years had a significantly increased risk of ERVs (6.2%, AOR: 1.49, 95% CI: 1.39–1.59). In comparison to patients with injury diagnoses (2.2%), those with noninjury diagnoses had a higher risk of ERVs (5.2%, AOR: 2.50, 95% CI: 2.33–2.70). Compared with patients initially treated at medical centers (3.7%), those initially treated at regional (4.5%, AOR: 1.28, 95% CI: 1.20–1.37) or district hospitals (4.5%, AOR: 1.38, 95% CI: 1.27–1.49) had significantly higher risks of ERVs. Among the 6740 patients with ERVs, 2622 (38.9%) returned to a different hospital, and these patients tended to be those aged 18 to 64 years and initially treated at district hospitals.

The risk of ERVs was associated with demographic characteristics and accreditation level of hospital. We noted a large proportion of patients with ERVs to a different hospital. The reason underlying this phenomenon warrants further investigations.

## INTRODUCTION

When patients return to the emergency department (ED) shortly after a previous visit, it is generally assumed that their initial evaluation or treatment was inadequate. Early return visits (ERVs) to the ED raise concerns regarding poor initial care, such as a missed diagnosis or inadequate treatment. One previous publication called these ERV patients “red-flag patients.” These patients were frequently dissatisfied and represented a medico-legal high-risk group. Many of the patients who sought legal action had presented to the ED more than once for the same condition.^1^

ERVs to the ED may cause a considerable increase in the ED workload. Many ERVs may be medically unnecessary; it is known that a substantial proportion of patients use the ED for nonemergent problems.^1–4^ One study on return visits to the ED of a hospital in Canada reported that of the 9935 ED visits 289 (2.9%) were return visits within 72 hours. Most unexpected return visits were categorized as low-acuity (45.3%), and most patients (88.6%) were treated in the ED and discharged home.^4^ Lerman and Kobernick reported that of the 64,336 ED visits during study period, 255 returned within 72 hours. Eighty-three (32.5%) of the returns were found to be avoidable with better patient education or medical care on the initial visit.^1^ Better patient education may minimize misuse of ED service and enable better care for those who really need it.

The reasons for return visits to the ED are complex and multifactorial. Previous reports have highlighted at least 4 potential categories of factors related to return visits, including the patient, disease, clinician, and health care system. Some factors, including increasing patient age,^5–7^ severity of the illness,^5,8^ disease progression or nonresolution,^9,10^ and junior or inexperienced clinicians,^11,12^ were reported to be associated with return visits. High ERV rates may be indicative of poor clinical care, systemic failures, and/or poor access to alternative primary care services.^13^

ERVs to the ED are an important quality indicator of the performance of individual physicians, EDs, and systems responsible for emergency care.^13,14^ However, the definitions of ERVs in previous studies were inconsistent. The majority of studies used a 72-hour window between initial and subsequent visits.^13^ In addition, some previous studies included all ERVs with both related and unrelated diagnoses^2,15,16^ and others included only ERVs with related diagnoses.^12^ Furthermore, most of the previous studies regarding ERV to the ED were hospital-based.^1,2,12,15,16^ Patients might initially present to the ED at 1 hospital and have their ERVs at another hospital, a situation that cannot be evaluated by a hospital-based study. As a result, hospital-based studies could be underestimating the actual scale of ERVs. ERVs to another hospital may have a significant impact on clinical performance and, to our knowledge, have not been evaluated before.

Using a nationally representative sample retrieved from the National Health Insurance Research database (NHIRD), this study aims to evaluate the proportion of ERVs to the ED within 3 days and to investigate the association between ERVs to the ED within 3 days and various characteristics related to patients, illness, physicians, and medical institution. The results from this study may serve as a benchmark for quality assurance in emergency care.

## MATERIALS AND METHODS

### Study Design and Data Source

This is a cross-sectional population-based study using data obtained from the NHIRD. Taiwan's National Health Insurance (NHI) is a ubiquitous public health insurance program initiated in 1995. NHI is managed under a governmental organization, the Bureau of NHI (BNHI), and it is funded through a combination of premiums and taxes.^17^ The NHI program enrolled about 96% of the Taiwanese population, and the BNHI had contracted with 97% of hospitals and clinics throughout the country by the end of 1996.^18^ In NHI system, patients have liberty to visit any medical institution of their choice and there are no referral requirements.^19^ In 1999, the BNHI began to release all claims data in electronic form to the public under the project of NHIRD. NHIRD is open to scientists for research purposes. For privacy protection, the data which can be used to identify patients or care providers are encrypted.^20^ NHIRD contains “cohort datasets” including claims data randomly sampled, in year 2000, 2005, and 2010, from all beneficiaries. The purpose of cohort datasets is to follow-up a representative group of the population longitudinally.^21^ In our study, we used longitudinal health insurance database 2010 (LHID2010). LHID2010 contains all registry and claim data of 1 million subjects randomly sampled in year 2010. The registration data of about 27.38 million people who were beneficiaries of the NHI program during the period of January 1, 2010 to December 31, 2010 were drawn for random sampling.^22^ New claim data of the cohort would be released every year. According to NHIRD, there was no significant difference in the gender distribution (χ^2^ = 0.067, df = 1, *P*-value = 0.796) between the patients in the LHID2010 and the original NHIRD.^23^

After ethical approval from the institution review board of Taipei City Hospital and the BNHI, the 2012 annual Ambulatory Care Expenditure by Visits (ACEV) file from LHID2010 was analyzed in this study. The ACEV provides information on the dates of the visits, up to 3 diagnoses initially coded by physicians, encrypted identification numbers (IDs) of the patients and attending physicians, the sexes and dates of birth of the patients, and the codes of medical facilities.^24^ In addition, the ACEV has codes for physician fees for emergency care that can be used to identify ED visits. For each ED visit there is a code to specify whether the patient is transferred to another medical facility for further care after ED treatment.^25^ Using encrypted individual personal IDs, we were able to interlink all the datasets. The Inpatient Expenditures by Admissions file provides information on hospitalizations. The Registry for Medical Personnel provides information on the ID, sex, and date of birth of each physician, and the Registry for Board-certified Specialists provides information on the specialty of each physician.^24^ Additionally, information on hospital accreditation levels was obtained from the Registry for Contracted Medical Facilities. Hospital accreditation has been implemented in Taiwan since 1978.^26^ Under Taiwan NHI, only medical care at accredited hospitals is eligible for NHI payment and most hospitals were accredited.^27^ There are 26 medical centers, 83 regional hospitals, and 370 district hospitals according to the data from Ministry of Health and Welfare in May, 2015.^28^

### Selection of the Study Participants and Outcome Measures

**FIGURE 1:**
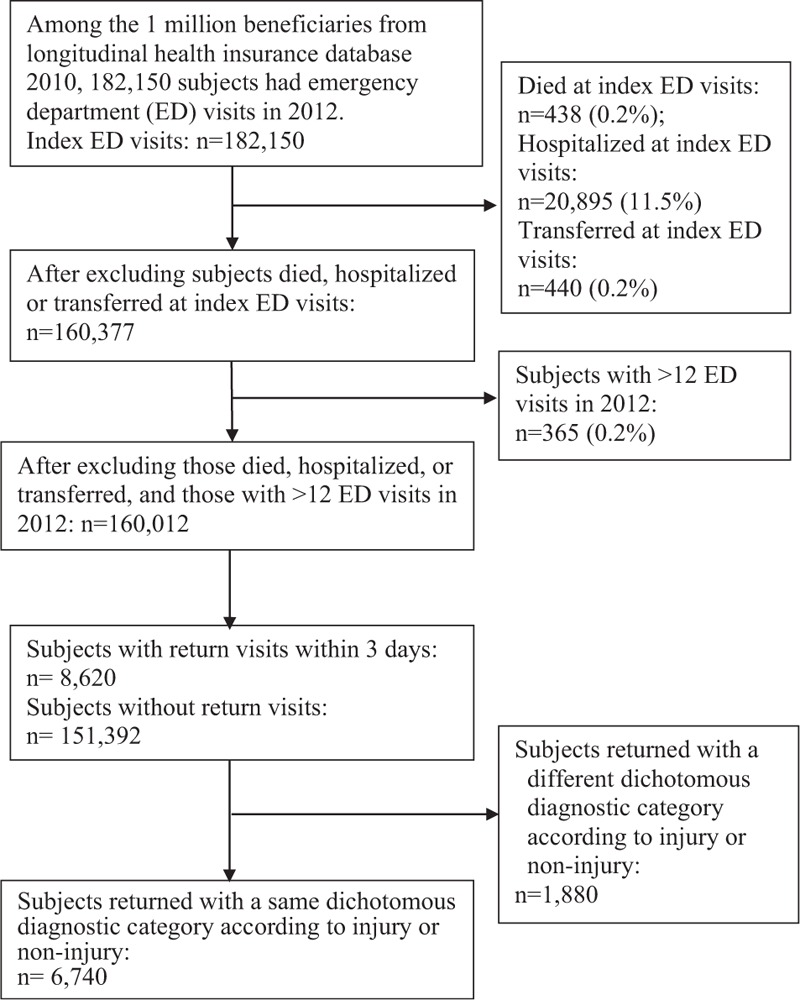
Selection of index emergency department visits and early return visits.

All ED visits in the year 2012 were analyzed for the proportion of and factors associated with ERVs to the ED within 3 days. An index ED visit was defined as the first ED visit, between January 15, 2012 and December 27, 2012, for any beneficiary (n = 182,150). An ERV to the ED was defined as a subsequent ED visit by the same patient on the same day or within 3 days following the index visit. Patients who died (n = 438, 0.2%), were hospitalized at index ED visits (n = 20,895, 11.5%), or were transferred to another hospital at index ED visits (n = 440, 0.2%), were excluded. Based on the concern that some frequent ED users might reflect a pattern of dependence on the ED as a source of care,^29,30^ subjects with >12 ED visits in 2012 were also excluded (n = 365, 0.2%). Fig. 1 illustrated the study patients enrollment and sequence of our analytical strategy.

The principal diagnoses (the first diagnosis on claim data) at index ED visits were categorized into 9 diagnostic categories: cancer-related diagnoses (International Classification of Diseases, version 9 code [ICD-9]: 140–208), endocrine disorders (ICD-9: 240–279), neurological diseases (ICD-9: 320–359), circulatory system diseases (ICD-9: 390–459), respiratory system diseases (ICD-9: 460–519), digestive system diseases (ICD-9: 520–579), symptoms and signs (ICD-9: 780–799), injuries (ICD-9: 800–999), and miscellaneous group.

To evaluate ERVs to the ED, we dichotomized the diagnostic categories into injury group and noninjury group and analyzed the proportion of and factors associated with ERVs to the ED within 3 days with a same dichotomous diagnostic category according to injury or noninjury. We also evaluated the proportion of and factors associated with hospitalization after ERVs to the ED, and ERVs to the ED of another hospital.

### Statistical Analysis

We first calculated the proportions of ERVs, hospitalization after ERVs, and ERVs to a different hospital. To investigate the independent effect of various characteristics, at the index visit, on ERVs to the ED we employed generalized estimation equation model after considering the clustering effect of ED patients. The generalized estimation equation model was also used to assess the independent effect of various characteristics on hospitalization after ERVs and on ERVs to the ED of another hospital. All statistical analyses were performed using SAS statistic software (version 9.3; SAS Institute, Cary, NC). A *P* < 0.05 was considered statistically significance.

## RESULTS

### Early Return Visits Within 3 Days With a Same Dichotomous Diagnostic Category According to Injury or Noninjury and Hospitalization After Return Visits

After excluding 1880 ERVs with a different dichotomous diagnostic category according to injury or noninjury there were 6740 ERVs with a same dichotomous diagnostic category (Fig. 1). The overall proportion of ERVs with a same dichotomous diagnostic category was 4.3% (Table 1). Male patients (4.3%) were more likely than female patients (4.2%) to have ERVs and had a statistically higher adjusted odds ratio (AOR = 1.10, 95% confidence interval [CI]: 1.04–1.16). Compared with patients aged 18 to 64 years (4.0%), those aged >64 years (6.2%, AOR: 1.49, 95% CI: 1.39–1.59) and those aged <6 years (4.9%, AOR: 1.17, 95% CI: 1.06–1.28) had increased risks of ERVs. In comparison to patients with injury diagnoses (2.2%), those with noninjury diagnoses had a higher risk of ERVs (5.2%, AOR: 2.50, 95% CI: 2.33–2.70).

**TABLE 1:**
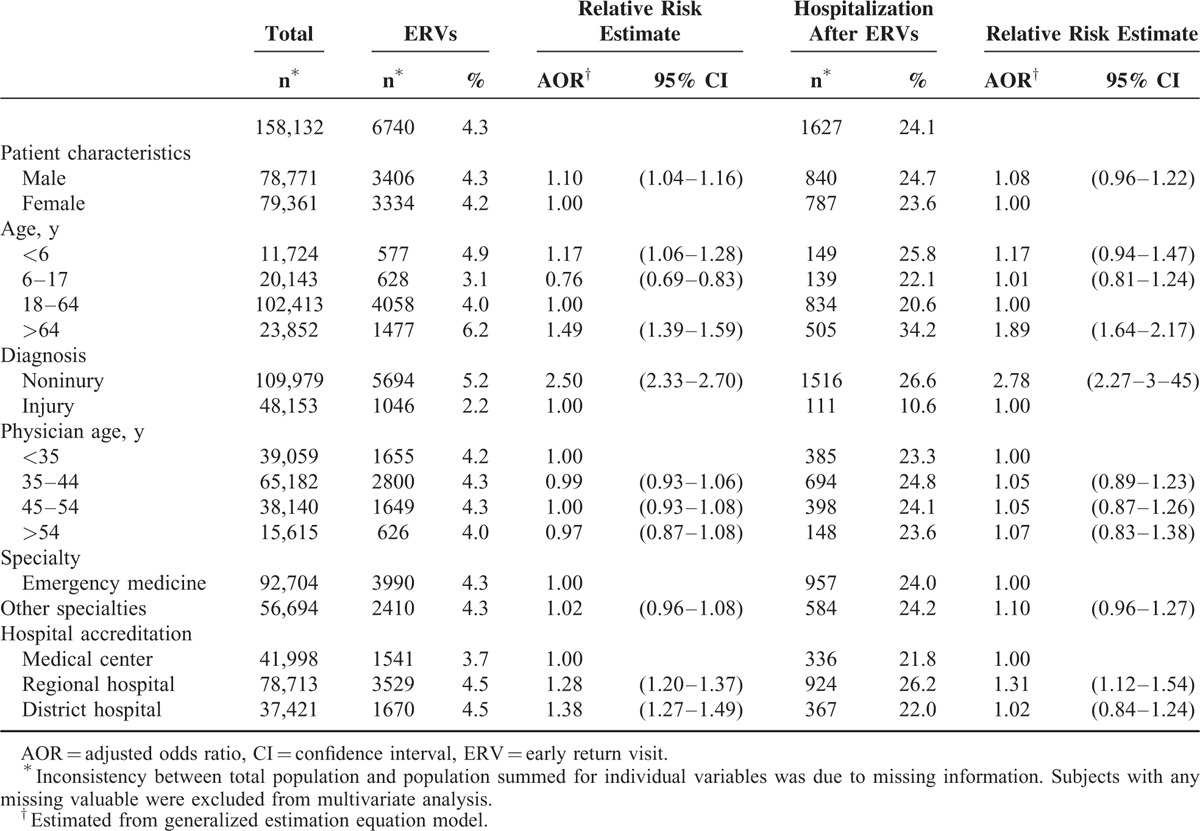
Proportions of and Predictors for ERVs Within 3 Days With a Same Dichotomous Diagnostic Category According to Injury or Noninjury and Hospitalization After Return Visits

With regard to physician characteristics, there was no statistically significant association between ERVs and physicians’ age, or specialty. The ERV proportions for patients initially treated at medical centers, regional hospitals, and district hospitals were 3.7%, 4.5%, and 4.5%, respectively. Compared with medical centers, regional hospitals (AOR: 1.28, 95% CI: 1.20–1.37), and district hospitals (AOR: 1.38, 95% CI: 1.27–1.49) had increased risks of ERVs.

The overall proportion of hospitalization after ERVs with a same dichotomous diagnostic category was 24.1% (Table 1), which was higher than the hospitalization proportion at index ED visits (11.5%, Fig. 1). Compared with patients aged 18 to 64 years (20.6%), those aged >64 years (34.2%, AOR: 1.89, 95% CI: 1.64–2.17) were more likely to be hospitalized after ERVs. In comparison to patients with injury diagnoses (10.6%), those with noninjury diagnoses (26.6%, AOR: 2.78, 95% CI: 2.27–3.45) had an increased risk of hospitalization after ERVs. Compared with subjects initially treated at medical centers (21.8%), those initially treated at regional hospitals (26.2%, AOR: 1.31, 95% CI: 1.12–1.54) had an increased risk of hospitalization after ERV.

### Early Return Visits to Another Hospital Within 3 Days With a Same Dichotomous Diagnostic Category According to Injury or Noninjury

ERVs to the ED of another hospital accounted for 38.9% of all ERVs within 3 days with a same dichotomous diagnostic category (Table 2). Compared with patients aged 18 to 64 years (42.6%), those aged <6 years (32.4%, AOR: 0.74, 95% CI: 0.60–0.91), those aged 6 to 17 years (35.5%, AOR: 0.78, 95% CI: 0.65–0.94), and those aged >64 years (32.8%, AOR: 0.68, 95% CI: 0.59–0.77) were statistically less likely to return to the ED of another hospital. Compared with subjects initially treated by physicians aged <35 years (36.8%), those initially treated by physicians aged >54 years had an increased risk of ERVs to the ED of another hospital (50.3%, AOR: 1.28, 95% CI: 1.05–1.57). In comparison to subjects initially treated at medical centers (31.9%), those initially treated at regional hospitals (35.5%, AOR: 1.18, 95% CI: 1.02–1.36) or district hospitals (52.5%, AOR: 2.23, 95% CI: 1.89–2.64) had increased risks of ERVs to the ED of another hospital.

**TABLE 2:**
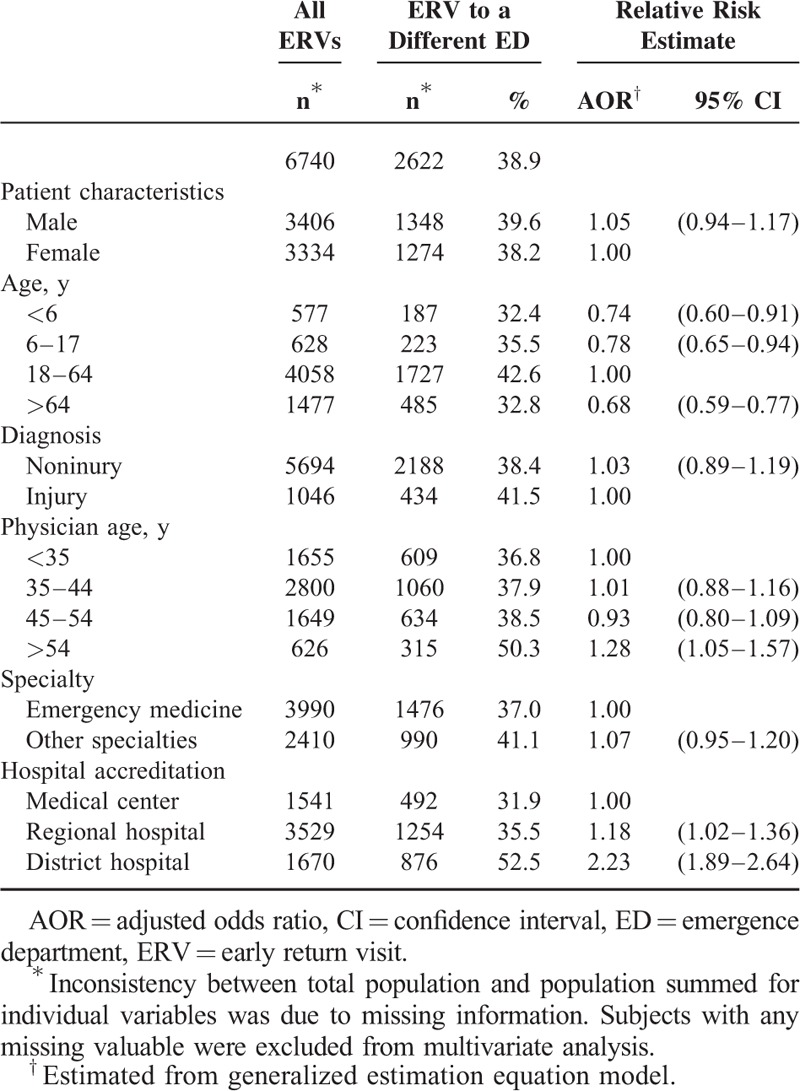
Proportions of and Predictors for ERVs to the ED of Another Hospital Within 3 Days With a Same Dichotomous Diagnostic Category According to Injury or Noninjury

## DISCUSSION

The analysis of the nationwide database offers a comprehensive overview of ERVs to the ED and information regarding the variation in ERV proportions among hospitals. This analysis avoids biases introduced from using data from only one or a few EDs. Furthermore, the large number of study subjects in our series made it possible to analyze more variables of interest, including patient, disease, physician, and hospital characteristics. In addition, we were able to evaluate ERVs to the ED of another hospital.

The international data suggest that an early ERV rate of approximately 3% is a reasonable estimation of the average global return visit rate.^13^ However, there is a large variation in the literature. The time span of ERVs may determine the rate of ERVs to the ED. A longer time span may increase ERV rate and may include patients who are either chronic disease patients, frequent attenders, or have an unrelated attendance. The majority of studies used a 72-hour window between initial and subsequent visits.^13^ In addition, some previous studies included all ERVs with both related and unrelated diagnoses,^2,15,16,31^ and others included only ERVs with related diagnoses.^12^ In our study, we used 3 days as a time span between initial ED visits and return ED visits to analyze ERVs with a same dichotomous diagnostic category according to injury or noninjury. We found that the overall proportion of ERVs within 3 days with a same dichotomous diagnostic category was 4.3% and ERVs to a different hospital accounted for 38.9%.

Several studies reported on ERV rates using a definition of return visits within 72 hours after the initial ED visits, with results ranging from 1.3% to 7.5%.^2,12,15,16,31^ Most of these studies included ERVs with both related and unrelated diagnoses. One retrospective cohort study of all nonfederal ED discharges in Florida and Nebraska reported a rate of all ED revisits, regardless of diagnoses, as up to 7.5%.^31^ One study reported monthly ERV rates ranging from 1.3% to 2.4% at a medical center in North Taiwan.^16^ Another study reported higher monthly rates ranging from 2.85% to 6.25% at a regional hospital in central Taiwan.^15^ Keith et al^2^ reviewed charts of all ED patients at a hospital in the United States of America and reported that 455 of 13,261 (3.4%) patients returned to the ED within 72 hours. One study in Singapore reported a return rate of 2% after excluding returns for unrelated diagnoses.^12^

Our study demonstrated that male patients have a higher risk of ERVs to EDs. One study on the ecology of medical care in Taiwan reported that, compared with women, a higher proportion of men (87.6/1000 vs 81.0/1000) received emergency services in 2005.^32^ Other studies found that women were possibly more health-conscious^33^ and that men usually seek medical help at a later stage of their illness.^34^ Furthermore, women may have a lower employment rate,^35^ providing them more time to visit physicians during office hours. In addition the differences in clinical presentations between men and women for some diseases may lead clinicians to misdiagnose or under-investigate one group. One study, on gender differences in presentation and diagnosis of chest pain in primary care reported that a significant higher number of women than men presented with chest pain to the general practitioners. However, chest wall syndrome, coronary heart disease, and psychogenic disorders accounted 48.6%, 13.0%, and 11.2%, respectively, of the final diagnoses for women and the corresponding figures for men were 44.0%, 17.2%, and 7.3%, respectively.^36^ The author argued that women rated their pain as more intense and used more affective words to describe their pain, both mechanisms that might contribute to a lower threshold to consult a general practitioner for further investigations.^37^ These differences in health-seeking behaviors as well as the differences in clinical presentations and underlying diseases might increase the risk of ERVs to the ED for male patients.

Consistent with previous studies, people aged >64 years had the highest risk of ERVs to EDs.^13^ A higher proportion of elderly people have chronic diseases and multiple comorbidities,^38^ which might increase the likelihood of requiring emergency services and having an ERV to the ED.^39^ Furthermore, a common disease may have an atypical course in elderly patients or might present with atypical manifestations^40–42^ that may result in a misdiagnosis or early release from the ED, prompting a return to the ED shortly after being discharged. In addition, previous studies disclosed that loneliness and insufficient family support to be associated with increased ED visits.^43–45^ Geller et al^43^ reported a significant correlation between loneliness score and total hospital ED visits. Hastings et al^44^ reported that among old people aged >64 years those who lived alone were more likely to visit the ED than those who lived solely with their spouse. Carret et al^45^ reported that among ED patients lack of social support is a risk factor for higher inappropriate ER use. Coe et al^46^ reported that older people without a family network were more likely to visit EDs. Data from Australia demonstrated that an increase in dual income families and increased geographic mobility of the workforce in the past 20 years might contribute to the fragmentation of extended family unit, compromising the capacity to support and care for older relatives.^47^ Loneliness and insufficient family or social support for old people might increase the risk of ERV in old people.

Illness characteristics are an important factor for ERVs, and the natural course of diseases may dictate when patients return.^13^ McCusker et al^7^ reported that a history of heart disease is a predicting factor for returns to EDs within 1 month among patients aged 65 years or older. Asthma and chronic obstructive airway disease accounted for a substantial proportion of ERVs for related complaints within 48 hours of discharge from the ED.^48^ In our study, compared with patients visiting EDs with injury diagnoses, those visiting EDs with noninjury diagnoses had a higher risk of ERVs to EDs.

Inconsistent with previous studies, which reported that junior or inexperienced clinicians were associated with ERVs,^11,12^ there was no significant association between ERVs and physician age in our study. With regarding to the association between ERVs and physician specialty, the Taiwan Society of Emergency Medicine was founded in 1994. Emergency medicine became a nationally recognized medical specialty, and a certification board was formed in Taiwan in 1997.^49^ Currently, a total of 1348 emergency medicine specialists are registered at the Ministry of Health and Welfare.^50^ Since there are more than 400 hospitals in Taiwan not all hospital EDs are equipped with emergency medicine specialists.^28^ Some ED patients are treated by physicians of other specialties. In our study, we did not observe significant association between ERV proportion and physician specialty.

Our study demonstrated that compared with medical centers, regional hospitals and district hospitals had increased risks of ERVs. Factors, including clinical experience, staff deficiencies, specialty support, and guideline adherence for emergency care can influence physicians’ decision-making processes. The ability of ED physicians to offer patients a correct diagnosis and adequate treatment at their initial ED presentation is based on the above factors and factors related to the patients and their illnesses.^13^ It is reasonable to speculate that ED physicians in hospitals with a higher accreditation level might have better specialty support and better access to continuing medical education to improve adherence to guidelines for emergency care. As a result, the risk of ERVs to EDs was lowest for medical centers and highest for district hospitals.

In our study, ERVs to EDs of other hospitals accounted for substantial proportions of all ERVs. Patients aged 18 to 64 years had the highest likelihood of returning to EDs of other hospital. One possible explanation is the mobility and health-seeking behavior of patients in this younger age group. These patients may choose an ED with better-trained physicians and better ancillary services, such as laboratory and radiology services. As a result, they had the highest risk of ERVs to EDs of other hospitals. On the contrary, a higher proportion of elderly people have chronic diseases^38^ that make them receive follow-up care at the same hospital, and thus they exhibit a lower risk of ERVs to EDs of other hospitals. Similarly, one previous study reported that approximately three-fourths of frequent ED use children are identified as having chronic conditions and they were more likely to have a primary pediatrician.^51^ As a result child patients were less likely to have ERVs to EDs of other hospitals.

Subjects initially treated at district hospitals had highest risks of ERV to EDs of other hospitals. The NHI program in Taiwan provides mandatory universal health insurance and comprehensive medical coverage to nearly all civilian Taiwanese residents. Patients only pay minimal user fees and copayments for ambulatory care services in hospital outpatient settings and EDs. In addition, patients have the freedom to use the ED at any medical institution when they have a medical emergency.^32^ Limited specialty support for emergency consultation and limited advanced equipment at district hospitals might increase the risk of ERVs to EDs of other hospitals for patients initially treated at EDs of district hospitals. In the United States of America, one statewide analysis on ED utilization in Massachusetts reported that 58% of frequent ED users used multiple EDs in fiscal year 2003.^52^ People may visit the ED of another hospital when their medical emergency is not addressed. ERVs to EDs of other hospitals may lead to underestimations of the actual scale of ERVs in hospital-based studies.

We used hospitalizations after ERVs as an index of disease severity. The overall rate of hospitalization after ERVs with a same dichotomous diagnostic category was 24.1%. Subjects aged >64 years were more likely to be hospitalized after ERVs, possibly because of reduced bodily function, atypical presentations, increased complexity of their diseases, and poor compliance with therapy.^53,54^ Our results were similar to that by Gabayan et al.^55^ In their study on factors associated with short-term bounce-back admissions, they reported that older age was associated with increased risks of hospitalization within 7 days following discharge from ED visits.

ERVs and ERVs with hospitalization demonstrate the need to improve ED and follow-up care. Comprehensive care with multidisciplinary resources, including specialized geriatric services and social workers for older ED patients, might be implemented to reduce the ERV rate and hospitalization rate after ERVs. Follow-up telephone calls are reported to be effective at reducing readmission rates and ED visits for recently hospitalized patients and have become routine in primary care settings.^56^ Telephone follow-ups may be used for some patients after ED discharge. These calls offer physicians a timely access to clarify discharge instructions and to evaluate condition changes in response to treatment.

## LIMITATIONS

First, using administrative database we were unable to obtain detailed information about presenting symptoms and clinical courses at ED visits. Representations for nonurgent follow-up visits are different to those due to deterioration. Second, we did not have information on some patient characteristics, such as socio-economic status, level of education, compliance with treatments, and confidence in their primary care, which are associated with ERVs and might confound the study results. Any discrepancy between the patients’ expectations of health care at EDs and the services really offered at initial ED visits might result in ERVs.^13^ In addition the degree of patients’ confidence in their primary care providers may affect the patient's decision for revisiting EDs following initial ED visits. Agarwal et al^57^ reported that the patient's perceptions of the effectiveness of and access to primary care were factors associated with a patient's decision to attend EDs. Previous studies reported that frequent ED users were more likely than less-frequent ED users to be poor or near poor,^29^ and a substantial proportion of frequent ED users were homeless or qualified for public assistance.^58^ In our study, we excluded subjects with more than 12 ED visits in 2012 based on the concern that some frequent ED users might reflect a pattern of dependence on the ED as a source of care.^29^ Exclusion of frequent ED users may lead to underestimations of ERVs although the number of frequent ED users excluded in our study is limited (0.2%, 365/182,150). Third, previous studies reported that mental disorders increased the risk of ED attendance. Hunt et al^59^ indicated that poor mental health increased the risk of ED visits. Sandoval et al^60^ stated that frequent ED visitors were much more likely to screen positively for depression. One study in Taiwan reported that among the 54,341 ED visits there were 13,196 (24.3%) and 2,952 (5.4%) patients with 2 and 3 concomitant diagnoses, respectively. The distribution of patient disease codes revealed that codes of mental disorders (ICD-9: 290–319) accounted for 2.4% of all patient disease codes.^61^ In our study, mental illness was categorized into the miscellaneous diagnostic category and the overall ERV rate for the miscellaneous diagnostic category was 3.1%. Fourth, in our study up to 38.9% of patients visited EDs of other hospitals and subjects initially treated at district hospitals had highest risks of ERVs to EDs of other hospitals. The difference in rates of ERVs to EDs of other hospitals between medical centers and district hospitals might come from the differences in subspecialty support and advanced equipment; however, we did not analyze whether patients presented to a hospital that was larger or smaller than that of the index presentation. In addition, we did not evaluate the potential role of geography in these representations. Further studies are needed to clarify these issues.

## CONCLUSIONS

The overall proportion of ERVs within 3 days with a same dichotomous diagnostic category according to injury or noninjury was 4.3% and ERVs to a different hospital accounted for 38.9% of all ERVs. The overall proportion of hospitalizations after ERVs was 24.1%. Male gender, age >64 years, initial ED visits that occurred at regional or district hospitals, increased the risk of having an ERV. Patients aged 18 to 64 years or who were initially treated at district hospitals were more likely to return to the ED of another hospital. Age >64 years were associated with hospitalization after ERVs.

## References

[1] Lerman B Kobernick MS Return visits to the emergency department. *J Emerg Med* 1987; 5:359–362. 3668198 10.1016/0736-4679(87)90138-7

[2] Keith KD Bocka JJ Kobernick MS Emergency Department revisits. *Ann Emerg Med* 1989; 18:964–968. 2764329 10.1016/s0196-0644(89)80461-5

[3] O’Dwyer F Bodiwala GG Unscheduled return visits by patients to the accident and emergency department. *Arch Emerg Med* 1991; 8:196–200. 1930505 10.1136/emj.8.3.196 PMC1285777

[4] Foran A Wuerth-Sarvis B Milne WK Bounce-back visits in a rural emergency department. *Can J Rural Med* 2010; 15:108–112. 20604996

[5] Abualenain J Frohna WJ Smith M The prevalence of quality issues and adverse outcomes among 72-hour return admissions in the emergency department. *J Emerg Med* 2013; 45:281–288. 23352864 10.1016/j.jemermed.2012.11.012

[6] Martin-Gill C Reiser RC Risk factors for 72-hour admission to the ED. *Am J Emerg Med* 2004; 22:448–453. 15520938 10.1016/j.ajem.2004.07.023

[7] McCusker J Cardin S Bellavance F Return to the emergency department among elders: patterns and predictors. *Acad Emerg Med* 2000; 7:249–259. 10730832 10.1111/j.1553-2712.2000.tb01070.x

[8] Nuñez S Hexdall A Aguirre-Jaime A Unscheduled returns to the emergency department: an outcome of medical errors? *Qual Saf Health Care* 2006; 15:102–108. 16585109 10.1136/qshc.2005.016618 PMC2464826

[9] Whiticar R Webb H Smith S Re-attendance to the emergency department. *Emerg Med J* 2008; 25:360–361. 18499822 10.1136/emj.2007.050617

[10] Pierce JM Kellerman AL Oster C "Bounces”: an analysis of short-term return visits to a public hospital emergency department. *Ann Emerg Med* 1990; 19:752–757. 2389858 10.1016/s0196-0644(05)81698-1

[11] Ross MA Hemphill RR Abramson J The recidivism characteristics of an emergency department observation unit. *Ann Emerg Med* 2010; 56:34–41. 20303200 10.1016/j.annemergmed.2010.02.012

[12] Kuan WS Mahadevan M Emergency unscheduled returns: can we do better? *Singapore Med J* 2009; 50:1068–1071. 19960161

[13] Trivedy CR Cooke MW Unscheduled return visits (URV) in adults to the emergency department (ED): a rapid evidence assessment policy review. *Emerg Med J* 2015; 32:324–329. 24165201 10.1136/emermed-2013-202719

[14] Lindsay P Schull M Bronskill S The development of indicators to measure the quality of clinical care in emergency departments following a modified-Delphi approach. *Acad Emerg Med* 2002; 9:1131–1139. 12414461 10.1111/j.1553-2712.2002.tb01567.x

[15] Wu CL Wang FT Chiang YC Unplanned emergency department revisits within 72 hours to a secondary teaching referral hospital in Taiwan. *J Emerg Med* 2010; 38:512–517. 18947963 10.1016/j.jemermed.2008.03.039

[16] Liaw SJ Bullard MJ Hu PM Rates and causes of emergency department revisits within 72 hours. *J Formos Med Assoc* 1999; 98:422–425. 10443066

[17] Lu JF Hsiao WC Does universal health insurance make health care unaffordable? Lessons from Taiwan. *Health Aff* 2003; 22:77–88. 10.1377/hlthaff.22.3.77 12757274

[18] Chang TLC Taiwan's 1995 health care reform. *Health Policy (Amsterdam, Netherlands)* 1997; 39:225–239. 10.1016/s0168-8510(96)00877-9 10165463

[19] Cheng SH Chiang TL The effect of universal health insurance on health care utilization in Taiwan. Results from a natural experiment. *JAMA* 1997; 278:89–93. 9214512 10.1001/jama.278.2.89

[20] Lin WH Li CY Wang WM Incidence of end stage renal disease among type 1 diabetes: a nationwide cohort study in Taiwan. *Medicine (Baltimore)* 2014; 93:e274. 25526457 10.1097/MD.0000000000000274 PMC4603135

[21] Chen TJ Chou LF Hwang SJ Patterns of ambulatory care utilization in Taiwan. *BMC Health Serv Res* 2006; 6:54. 16672073 10.1186/1472-6963-6-54 PMC1468399

[22] Lee CW Liao CH Lin CL Depression and risk of venous thromboembolism: a population-based retrospective cohort study. *Psychosom Med* 2015; 77:591–598. 25984821 10.1097/PSY.0000000000000193

[23] National Health Insurance Research Database. Taiwan Available at. http://nhird.nhri.org.tw/en/Data_Subsets.html [Accessed July 27, 2015].

[24] Chen CC Wu LC Li CY Non-adherence to antibiotic prescription guidelines in treating urinary tract infection of children: a population-based study in Taiwan. *J Eval Clin Pract* 2011; 17:1030–1035. 20738469 10.1111/j.1365-2753.2010.01469.x

[25] Code Format. National Health Insurance Research Database, Taiwan Available at http://nhird.nhri.org.tw/file_date/en_codeformat.pdf [Accessed July 28, 2015].

[26] Wung CH The reform of the hospital accreditation system in Taiwan. *World Hosp Health Serv* 2008; 44:14–15. 18549028

[27] Huang CI Wung C Yang CM Developing 21st century accreditation standards for teaching hospitals: The Taiwan experience. *BMC Health Serv Res* 2009; 9:232. 20003505 10.1186/1472-6963-9-232 PMC2801490

[28] Statistics of Ministry of Health and Welfare. Available at http://www.mohw.gov.tw/cht/DOS/Statistic.aspx?f_list_no = 312&fod_list_no = 1828 [Accessed on July 27, 2015].

[29] Zuckerman S Shen YC Characteristics of occasional and frequent emergency department users: do insurance coverage and access to care matter? *Med Care* 2004; 42:176–182. 14734955 10.1097/01.mlr.0000108747.51198.41

[30] Ruger JP Richter CJ Spitznagel EL Analysis of costs, length of stay, and utilization of emergency department services by frequent users: implications for health policy. *Acad Emerg Med* 2004; 11:1311–1317. 15576522 10.1197/j.aem.2004.07.008

[31] Rising KL Victor TW Hollander JE Patient returns to the emergency department: the time-to-return curve. *Acad Emerg Med* 2014; 21:864–871. 25154879 10.1111/acem.12442

[32] Shao CC Chang CP Chou LF The ecology of medical care in Taiwan. *J Chin Med Assoc* 2011; 74:408–412. 21962249 10.1016/j.jcma.2011.08.005

[33] Pinkhasov RM Wong J Kashanian J Are men shortchanged on health? Perspective on health care utilization and health risk behavior in men and women in the United States. *Int J Clin Pract* 2010; 64:475–487. 20456194 10.1111/j.1742-1241.2009.02290.x

[34] Banks I No man's land: men, illness, and the NHS. *BMJ* 2001; 323:1058–1060. 11691768 10.1136/bmj.323.7320.1058 PMC1121551

[35] Directorate-General of Budget. Accounting and statistics, executive yuan, R.O.C. (Taiwan) http://www.stat.gov.tw/public/Attachment/081918204771.doc [Accessed December 17, 2010].

[36] Bösner S Haasenritter J Hani MA Gender differences in presentation and diagnosis of chest pain in primary care. *BMC Fam Pract* 2009; 10:79. 20003406 10.1186/1471-2296-10-79 PMC2801475

[37] D’Antono B Dupuis G Fleet R Sex differences in chest pain and prediction of exercise-induced ischemia. *Can J Cardiol* 2003; 19:515–522. 12717487

[38] Wolff JL Starfield B Anderson G Prevalence, expenditures, and complications of multiple chronic conditions in the elderly. *Arch Intern Med* 2002; 162:2269–2276. 12418941 10.1001/archinte.162.20.2269

[39] Gijsen R Hoeymans N Schellevis FG Causes and consequences of comorbidity: a review. *J Clin Epidemiol* 2001; 54:661–674. 11438406 10.1016/s0895-4356(00)00363-2

[40] Tresch DD Atypical presentations of cardiovascular disorders in the elderly. *Geriatrics* 1987; 42:31–46. 3653693

[41] Fox RA Atypical presentation of geriatric infections. *Geriatrics* 1988; 43:58–68. 3360329

[42] O’Dell C Atypical presentations of neurological illness in the elderly. *Geriatrics* 1988; 43:35–37. 3335335

[43] Geller J Janson P McGovern E Loneliness as a predictor of hospital emergency department use. *J Fam Pract* 1999; 48:801–804. 12224678

[44] Hastings SN George LK Fillenbaum GG Does lack of social support lead to more ED visits for older adults? *Am J Emerg Med* 2008; 26:454–461. 18410815 10.1016/j.ajem.2007.07.005

[45] Carret ML Fassa AG Kawachi I Demand for emergency health service: factors associated with inappropriate use. *BMC Health Serv Res* 2007; 7:131. 17705873 10.1186/1472-6963-7-131 PMC2034385

[46] Coe RM Wolinsky FD Miller DK Elderly persons without family support networks and use of health services: a follow-up report on social network relationships. *Res Aging* 1985; 7:617–622. 4095383 10.1177/0164027585007004007

[47] Lowthian JA Curtis AJ Cameron PA Systematic review of trends in emergency department attendances: an Australian perspective. *Emerg Med J* 2011; 28:373–377. 20961936 10.1136/emj.2010.099226

[48] Wong TW Lam KW Reattendance audit in an inner-city emergency department. *J Accid Emerg Med* 1994; 11:213–217. 7894804 10.1136/emj.11.4.213 PMC1342448

[49] Taiwan Society of Emergency Medicine. http://www.sem.org.tw/tsem/en/index_new.php [Accessed on July 30, 2015].

[50] Annual report at Taiwan Society of Emergency Medicine. http://www.sem.org.tw/tsem/en/en_annual.php [Accessed July 30, 2015].

[51] Yamamoto LG Zimmerman KR Butts RJ Characteristics of frequent pediatric emergency department users. *Pediatr Emerg Care* 1995; 11:340–346. 8751167 10.1097/00006565-199512000-00003

[52] Fuda KK Immekus R Frequent users of Massachusetts emergency departments: a statewide analysis. *Ann Emerg Med* 2006; 48:9–16. 16781915 10.1016/j.annemergmed.2006.03.001

[53] Hu KW Lu YH Lin HJ Unscheduled return visits with and without admission post emergency department discharge. *J Emerg Med* 2012; 43:1110–1118. 22674038 10.1016/j.jemermed.2012.01.062

[54] McCusker J Ionescu-Ittu R Ciampi A Hospital characteristics and emergency department care of older patients are associated with return visits. *Acad Emerg Med* 2007; 14:426–433. 17369450 10.1197/j.aem.2006.11.020

[55] Gabayan GZ Asch SM Hsia RY Factors associated with short-term bounce-back admissions after emergency department discharge. *Ann Emerg Med* 2013; 62:136–144. 23465554 10.1016/j.annemergmed.2013.01.017 PMC3720678

[56] Menchine M Oberfoell S Schriger D Improving telephone follow-up for patients discharged from the emergency department: results of a randomized controlled trial. *Acad Emerg Med* 2013; 20:456–462. 23672359 10.1111/acem.12128

[57] Agarwal S Banerjee J Baker R Potentially avoidable emergency department attendance rates: interview study of patients’ reasons for attendance. *Emerg Med J* 2012; 29:e3. 22205782 10.1136/emermed-2011-200585

[58] Malone RE Whither the almshouse? Overutilization and the role of the emergency department. *J Health Polit Policy Law* 1998; 23:795–832. 9803363 10.1215/03616878-23-5-795

[59] Hunt KA Weber EJ Showstack JA Characteristics of frequent users of emergency departments. *Ann Emerg Med* 2006; 48:1–8. 16781914 10.1016/j.annemergmed.2005.12.030

[60] Sandoval E Smith S Walter J A comparison of frequent and infrequent visitors to an urban emergency department. *J Emerg Med* 2010; 38:115–121. 18462906 10.1016/j.jemermed.2007.09.042

[61] Yang NP Lee YH Lin CH Utilization of and direct expenditure for emergency medical care in Taiwan: a population-based descriptive study. *J Epidemiol* 2009; 19:41–48. 19164870 10.2188/jea.JE20080042 PMC3924095

